# Climate change, wildfire, and vegetation shifts in a high-inertia forest landscape: Western Washington, U.S.A.

**DOI:** 10.1371/journal.pone.0209490

**Published:** 2018-12-20

**Authors:** Joshua S. Halofsky, David R. Conklin, Daniel C. Donato, Jessica E. Halofsky, John B. Kim

**Affiliations:** 1 Washington State Department of Natural Resources, Olympia, Washington, United States of America; 2 Oregon Freshwater Simulations, Portland, Oregon, United States of America; 3 University of Washington, School of Environmental and Forest Sciences, Seattle, Washington, United States of America; 4 U.S. Department of Agriculture Forest Service, Pacific Northwest Research Station, Corvallis, Oregon, United States of America; Ecole Pratique des Hautes Etudes, FRANCE

## Abstract

Future vegetation shifts under changing climate are uncertain for forests with infrequent stand-replacing disturbance regimes. These high-inertia forests may have long persistence even with climate change because disturbance-free periods can span centuries, broad-scale regeneration opportunities are fewer relative to frequent-fire systems, and mature tree species are long-lived with relatively high tolerance for sub-optimal growing conditions. Here, we used a combination of empirical and process-based modeling approaches to examine vegetation projections across high-inertia forests of Washington State, USA, under different climate and wildfire futures. We ran our models without forest management (to assess inherent system behavior/potential) and also with wildfire suppression. Projections suggested relatively stable mid-elevation forests through the end of the century despite anticipated increases in wildfire. The largest changes were projected at the lowest and uppermost forest boundaries, with upward expansion of the driest low-elevation forests and contraction of cold, high-elevation subalpine parklands. While forests were overall relatively stable in simulations, increases in early-seral conditions and decreases in late-seral conditions occurred as wildfire became more frequent. With partial fire suppression, projected changes were dampened or delayed, suggesting a potential tool to forestall change in some (but not all) high-inertia forests, especially since extending fire-free periods does little to alter overall fire regimes in these systems. Model projections also illustrated the importance of fire regime context and projection limitations; the time horizon over which disturbances will eventually allow the system to shift are so long that the prevailing climatic conditions under which many of those shifts will occur are beyond what most climate models can predict with any certainty. This will present a fundamental challenge to setting expectations and managing for long-term change in these systems.

## Introduction

In western North America, climate change is likely to result in greater area burned as conditions that promote wildfire become more common [1, 2]. If disturbances are the catalyst for large vegetation shifts with climate change [3, 4], opportunities for such shifts are potentially greater under higher disturbance frequencies, which could provide more opportunities for the climatically-sensitive regeneration phase to interact with changing environmental conditions [5]. However, these opportunities may be less frequent in forests with infrequent disturbance regimes where disturbance intervals tend to be measured in centuries rather than decades (e.g., maritime and subalpine temperate conifer forests [6–9]), and large patches of stand-replacement fire (10^3^−10^5^ ha) are relatively common and within normal system behavior. Such forests can be described as having greater ‘landscape inertia’ because of longer disturbance-free periods, fewer broad-scale regeneration opportunities, and long-lived tree species that, in mature form, can tolerate suboptimal conditions for long time periods [10].

A key example of a high-inertia forest landscape occurs in the Pacific Northwestern United States. Like much of western North America, this region is already experiencing the effects of climate change, including higher temperatures [11], reduced snowpack [12], transitions from snow- to rain-dominant watersheds [13], decreased mountain precipitation [14], and lower summer streamflows [15]. Projections for the future suggest temperature increases (of 0.5 to 5.2°C by 2100; [16]), decreased growing season precipitation (by as much as 30% by late century; [16]), and increased wildfire area burned [4, 17]. These changes will likely impact the structure, composition, and function of ecosystems in the region, but such impacts may differ with regional variations in disturbance frequency and associated landscape inertia.

Long-term climate change impacts in infrequently disturbed forests are highly uncertain, both in terms of magnitude and timing. Being generally wetter and more productive than other forests, these systems could be partially buffered, or even serve as refugia, from global climatic trends, at least compared to drier, fire-prone forests [18–20]. This could serve to reduce the overall magnitude of vegetation changes. When changes do occur, however, the long intervals between disturbances, during which time climate conditions could change substantially, could set the stage for larger, relatively abrupt vegetation shifts [3, 21, 22].

Despite the potential importance of landscape inertia when assessing future change, its incorporation in vegetation projections under a changing climate has been more limited in high inertia and long-interval systems [23]. Common modeling approaches such as empirical species distribution models (SDMs) [24, 25] and process-based dynamic global vegetation models (DGVMs) [26] typically project vegetation shifts with climate without incorporating the potential tolerance of mature vegetation to climatic shifts. Other models, called state-and-transition simulation models (STSMs, [27]), can probabilistically include natural disturbances, vegetation dynamics and management in a single modeling environment at either coarser or finer scales than DGVMs (depending on the simulation objectives, existing knowledge of the processes being modeled, and data availability), and approaches have been developed recently to include shifts in vegetation with climate and disturbance regimes [28, 29]. Increasingly, approaches are being developed that incorporate elements of both empirical and mechanistic models [24, 25, 30, 31].

Building on methods developed for a frequent-fire system [29], we utilized a model approach to examine the potential influence of wildfire and landscape inertia on climate-related vegetation changes in an infrequently disturbed forest region. The methodology we used allows the *potential* distribution of vegetation zones to change incrementally with climate, but only allows *actual* shifts to occur following a severe disturbance (i.e., when landscape inertia is broken). Further, the approach alters fire occurrence from a deterministic (DGVM) to a more realistic stochastic process (STSMs). Consequently, there was always a probability that wildfire may not occur in a given parcel or year, potentially resulting in fewer opportunities for wildfires to catalyze vegetation change. Through the lens of landscape inertia, our specific objectives were to characterize:

1. Possible shifts in fire regimes with climate change using both a DGVM and climate-informed STSMs (cSTSMs).
2. Possible 21^st^-century vegetation change using both a DGVM and cSTSMs.
3. How changing climate and fire regimes may affect the abundance of early-seral (post-disturbance) and late-seral (mature/old-growth) structural conditions, two successional stages of high conservation value and management concern [32, 33].
4. The above cSTSM wildfire and vegetation trends with and without basic fire management (i.e., fire suppression success).

Outside of reducing area burned to represent fire suppression, our overall objectives did not include the influence of vegetation management (a separate, extensive analysis of its own) but rather to project the likely climate- and disturbance-driven template upon which management will operate in the coming century. Our results therefore reflect the intrinsic potential of a high-inertia forest, in the absence of human intervention other than fire suppression.

## Methods

### Study area

**Fig 1:**
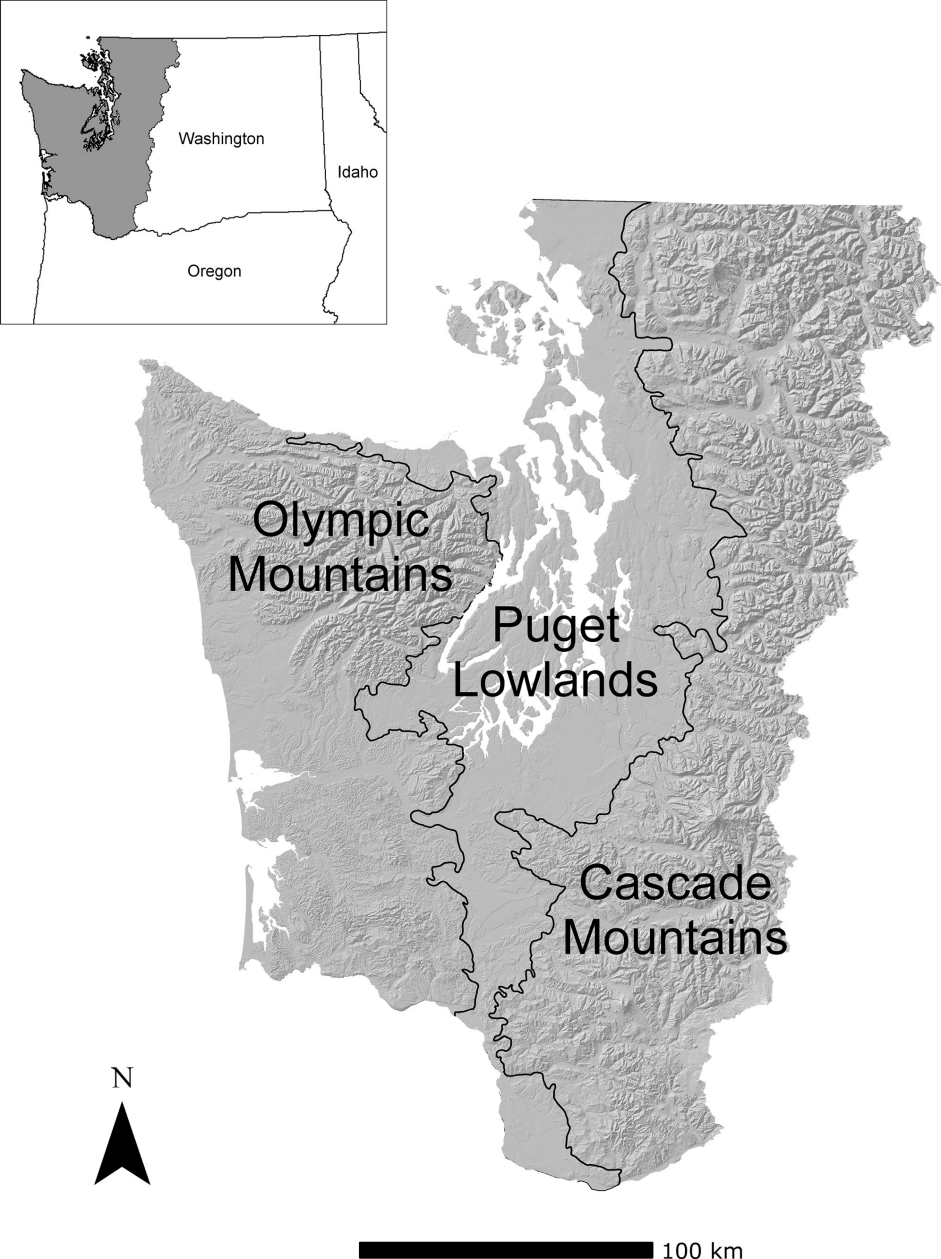
The study area covered by both the MC2 dynamic global vegetation model and the state-and-transition simulation models.

The study region covers approximately 5.26 million ha of forest west of the Cascade Mountain Crest in Washington State, USA (Fig 1). Elevation in the region ranges from sea level to 4392 m at the peak of Mount Rainier in the Cascade Mountains. The temperate maritime climate of western Washington is characterized by annual precipitation ranging from 500 to 5,000 mm. Coastal areas receive 3,000 to 5,000 mm of precipitation per year, while the crest of the Olympic Mountains receives >6,000 mm of precipitation per year, making it the wettest location in the conterminous United States [34]. In contrast, the northeastern portion of the Olympic Peninsula in the rain shadow of the Olympic Mountains and portions of the Puget Lowlands, are characterized by a drier climate, with rainfall as low as 500 mm per year at lower elevations [35].

Outside of the Puget Lowlands and Olympic Mountain rain shadow (Fig 1), fire was historically infrequent and large patches of stand-replacing fire were a major component of the fire regime (although most fires may be considered mixed-severity at the scale of the entire fire footprint). Fire rotations typically vary from 200 years in the highest elevation subalpine forests, to over 1,000 years along the Washington coast [8]. Drier forests found in the Puget Lowlands and Olympic Mountains rain shadow also have 200-year average fire rotations [8, 35], but historically experienced mixed-severity wildfire which also included more low severity wildfire than occurred in the rest of the study region. Across all of western Washington, we estimate the average natural fire rotation to be approximately 500 years based on forest zone area and average fire rotation values from the literature [8, 35].

### Climate scenarios

We used the 2010 version of the Parameter-elevation Relationships on Independent Slopes Model (PRISM) [36] for historical climate data [37] to calibrate the MC2 DGVM. This historical dataset is available on a 30-arcsec grid (~800m) and spans the years 1895–2009. For future climate projections, we obtained a subset of global circulation model (GCM) output prepared for the Intergovernmental Panel on Climate Change Fifth Assessment Report under representative concentration pathway 8.5, a scenario representing comparatively high greenhouse gas concentrations in absence of climate change mitigation policies [38]. We selected three GCMs for our analysis: CSIRO_MK360 RCP 8.5, HadGEM2_ES RCP 8.5, and NORESM1 RCP 8.5, hereafter referred to as CSIRO, HadGEM, and NorESM, respectively. We selected this subset of GCMs because they demonstrate relatively lower bias in the Pacific Northwest [39] and also span a range of future climate conditions in terms of average annual precipitation and temperature (Table 1). Seasonally, projected temperature increases are greater in the summer than winter while precipitation increases in winter and decreases during the summer months. The monthly values of four climate variables generated by each GCM were statistically downscaled to a 30-arcsec grid (~800) using the delta method [40]. These four variables were: precipitation, the monthly means of diurnal extreme temperatures, and a measure of atmospheric water content. The downscaled variables were used as input into a DGVM.

**Table 1:** Changes in modal temperature and precipitation values projected by three global circulation models (GCMs) for the study area.

	Global circulation model		
	CSIRO	HadGEM	NorESM
Temperature change relative to 1980–2009 (°C)			
2010–2039	+1.8	+1.0	+0.9
2040–2069	+2.8	+3.7	+2.1
2070–2099	+4.9	+5.9	+3.9
Precipitation change relative to 1980–2009 (mm)			
2010–2039	+31.6	-21.4	+229.6
2040–2069	-81.7	+30.3	+131.0
2070–2099	+179.3	-138.0	+242.0

CSIRO, HadGEM and NorESM respectively represent the CSIRO_MK360 RCP 8.5, HadGEM2_ES RCP 8.5, and NORESM1 RCP 8.5 GCMs.

### MC2 dynamic global vegetation model

#### Integration of species distribution models into MC2

**Fig 2:**
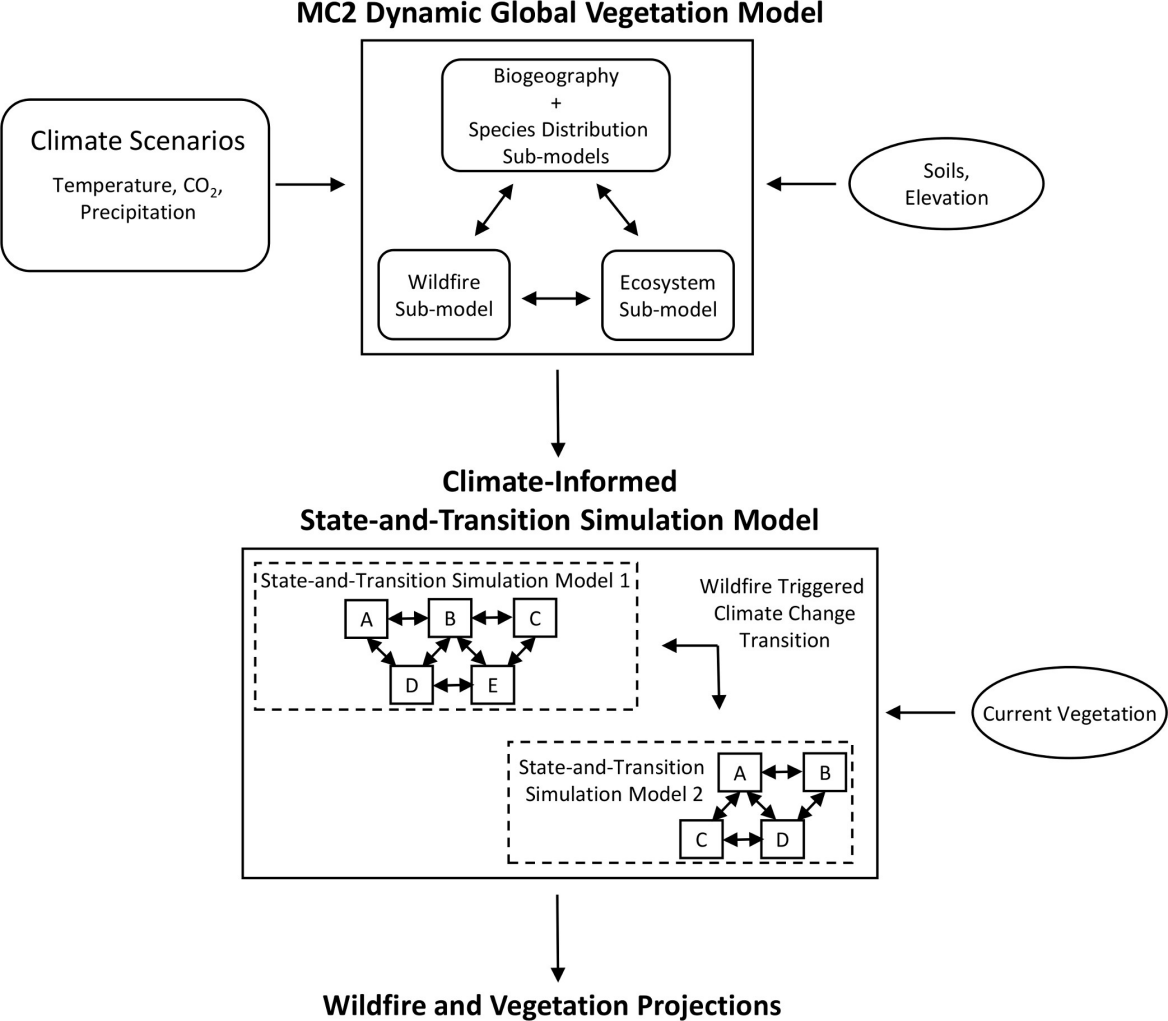
Conceptual overview of modeling process. Ellipses represent model input and rectangles represent models. Unbounded text indicates model outputs. In step1, future climate scenario and biogeographic information are input into the MC2 Dynamic Global Vegetation Model. Within MC2, three sub-models typically interact to project potential future vegetation, wildfire, and carbon, among other output. For this analysis, we included a species distribution model of forest zones within the biogeography sub-model. In step 2, MC2 wildfire and forest zone projections for each climate scenario are analyzed to develop forest transition average probabilities and both wildfire and forest zone trends. In step 3, the MC2-derived trends and probabilities are used to develop climate-informed state-and-transition simulation models (cSTSMs) for each climate scenario. Within a cSTSM, area is permitted to shift across previously developed state-and-transition simulation models (two simplified example models are shown) following a stand-replacing disturbance (i.e., when landscape inertia is broken). Figure adapted from [46].

MC2 is a DGVM that uses climate data to drive fundamental ecological processes such as plant competition for nutrients, water and light, and carbon and water uptake and losses [41]. MC2 is comprised of three sub-models: an ecosystem model, a biogeography model, and a wildfire model (see Step 1 in Fig 2). The ecosystem sub-model is based on the CENTURY Soil Organic Matter Model [42], and uses climate, soil, elevation and latitude to simulate net primary productivity of vegetation, and respiration and assimilation of carbon, to major ecosystem pools. The biogeography sub-model uses climate and ecosystem carbon pool and flux quantities simulated by the CENTURY sub-model to classify each grid cell into one of five possible biomes (forests, woodlands, shrublands, grasslands, and deserts) and then further classifies each grid cell into a plant lifeform (e.g., maritime needleleaf forest, temperate needleleaf forest, or temperate warm mixed forest). Notably, MC2 classifies the entire western Washington study area as forest biome under all future projections. The wildfire sub-model [43] incorporates moisture content of live and dead fuels (from soil moisture calculated in the ecosystem model), and live and dead fuel bed characteristics (1-h, 10-h, 100-h, and 1000-h fuels derived from the biogeography model), to simulate the initiation and behavior of a wildfire event. Fire events are initiated when region-specific fuel buildup thresholds are exceeded and fine fuel moisture drops below region-defined thresholds. Sources of ignition are assumed to always be available. Fire spread is not spatially explicit between 800-m simulation cells. Rather, the number of years since the last fire event divided by forest zone fire rotations are used to estimate the fraction of a cell annually burned.

#### Integration of species distribution models into MC2

In this study we replaced the MC2 biogeography ruleset for projecting a coarse-scale plant lifeform classification with species distribution models (SDMs), resulting in output of finer vegetation groups often used by land managers. Specifically, we adapted results from gradient modeling conducted by [44] to have MC2 classify grid cells into potential natural vegetation zones, hereafter referred to as forest zones. Henderson et al. [44] defined forest zones as the plant community or the set of communities capable of occupying a location absent disturbance or management. Eight input data layers were used to develop the forest zone model: aspect, cold air drainage, elevation, fog effect, mean air temperature at sea level, precipitation at sea level, shortwave radiation, and topographic moisture. By regressing thousands of field observation data on these eight data layers, [44] developed SDMs, identifying occupied environmental niches for different forest zones. The eight environmental variables were available as 90-m grids, but we aggregated them to 800 m to match the resolution of the climate input grids. Mean annual temperature at sea level and precipitation at sea level were calculated annually within MC2 from elevation and annual temperature and precipitation data provided by each GCM. We then applied ecoregion-specific regression equations developed by [44] to project future forest zones.

#### MC2 model calibration and projections

Western Washington was divided into three regions for MC2 calibration based on Level III Ecoregion boundaries [45], which represent a combination of biotic, abiotic, terrestrial and aquatic ecosystem components more similar to each other than to other ecoregions. Simulations for each ecoregion were run independently. Once historical vegetation was determined from the forest zone model for each region (S1 Table), we assigned pre-European historical fire rotations to each forest zone (see S2 Table for fire rotation values). These historical wildfire values were derived from the literature [8, 35] and are also the values used as wildfire probabilities in our STSMs (described later). The historical values do not incorporate fire suppression effects, and we did not include fire suppression in future MC2 projections.

As in other studies [29, 46, 47], prior to running the model under future climate scenarios, MC2 was run with historical climate data to ensure the model could reproduce historical conditions. MC2 was iteratively run to determine parameter values that produced vegetation fractions similar to historical vegetation proportions projected by [44], wildfire rotations similar to estimated historical rotations from the literature [8, 35], and MC2-derived NPP values similar to [48]. All MC2 simulations also included the default model CO_2_ fertilization effect (β = 0.25), thereby assuming some level of increase in productivity efficiency with greater atmospheric carbon dioxide levels [49].

Once calibrated, we incorporated temperature and precipitation projections from CSIRO, HadGEM, and NorESM climate futures into MC2 to project possible changes in vegetation and wildfire with climate change. We used these MC2 projections to develop climate change transitions for our STSMs (further discussed below).

### State-and-transition simulation models

We modeled landscape dynamics using probabilistic state-and-transition simulation models (STSMs, [27]) previously developed through the Integrated Landscape Assessment Project [50]. With STSMs, a continuum of conditions is typically grouped into discrete classes known as states. Transitions, representing agents of change in a STSM, link modeled states to each other. An individual state can have multiple transitions, where each transition can result in a different condition, or end state (Fig 2). Following [29, 50], we represented current forest structure and species information in our STSMS using a consistent ruleset based on dominant tree cover species, tree diameter, percent canopy cover, and canopy layers. Post-disturbance conditions were also included, representing surviving live trees of specific diameter ranges in addition to standing snags and downed wood. The number of states within our STSMs varied from seven to ~40 depending on vegetation type (models can be accessed through DRYAD). States were connected through transitions representing natural disturbances or succession. Primary stand-replacing disturbances in the model were episodic wind events and wildfire. Wind events were restricted to low and mid-elevation forests near the coast and occurred every 35 years, on average, based on the frequency of past events since the 1880s [8]. We developed wildfire estimates of fire rotations from the literature, assuming a predominantly stand-replacing fire regime in all forest zones other than the Douglas-fir zone, which we characterized as a mixed-severity fire regime [8, 35].

STSMs can be run both spatially and non-spatially [27]. We ran our models non-spatially, partly because there is a dearth of information on stand-replacing wildfire patch-size distributions due to the rarity of the disturbance, and partly because we sought to assess only broad-scale regional trends in forest vegetation. Wildfire was consequently a non-contagion event, where disturbance occurrence depends on probabilistic ‘rolls of the dice’. But as further discussed below, the probability of wildfire could be annually increased or decreased for a given climate future depending on MC2 wildfire projections.

### Climate-informed STSMs (cSTSMs)

We selected eight previously-developed STSMs representing all forest types in Washington State that were also modeled by MC2. Prior to this analysis, an STSM assigned to a given location in our study area remained fixed through time even with changes in climate. An earlier effort, resulting in methods used in this analysis [29], developed an approach to identify STSM-to-STSM climate change transitions informed by MC2 (Fig 2), thereby enabling area to move across STSMs with climate changes. We refer to these climatically linked STSMs as cSTSMs. While MC2 can generate spatial output, our approach relies on tallying information from individual pixels in the study area to generate regional tabular vegetation and wildfire data (Step 2 in Fig 2). Because our STSMs are non-spatial, we used this tabular information to develop two sets of region-wide STSM values: 1) average climate change transition probabilities of vegetation shifts, and 2) random sequences of inter-annual climate trends in vegetation shifts and wildfire.

Using the R environment for statistical computing (package:MC2toPath) and methods developed by [29], we developed average STSM climate change probabilities from the MC2 output. For a given climate future, we calculated the annual amount of area that moved from each MC2 forest zone to any other MC2 forest zone across the entire simulation period of 2010–2100. These annual forest zone area shifts were then divided by the total initial forest zone area, resulting in a time-series of annual proportion of a forest zone transitioning to another forest zone. Averaging the time-series of annual proportions for the entire future period yielded the average probability a forest zone would transition to another forest zone for a given climate future between 2010–2100. In theory, our method could result in the derivation of a climate change probability for each of our forest zones transitioning to each of the other seven forest zones. In practicality, we only selected those transitions affecting at least 1% of the landscape during the 2010–2100 period for a given climate future to capture the dominant possible changes in forests. These average probabilities became the climate change transitions in our cSTSMs (Step 3 in Fig 2). The transitions were added to the post-disturbance and grass/forb states in each STSM, assuming climate-related forest change is most likely following a stand-replacing disturbance (e.g., when landscape inertia is broken).

While our climate change transitions capture the average probability of a vegetation shift between 2010 and 2100, these averages could mask inter-annual variability of vegetation movement when the climate is more or less conducive to vegetation change. Like other studies [29, 46], we therefore developed inter-annual multipliers, or trend multipliers, to account for changes in the magnitude of vegetation movement with time. For a given climate scenario and forest zone transition, the previously calculated time-series data of annual proportion was divided by the average transition probability to normalize the data and develop the trend multipliers. Inter-annual multipliers <1 decreased the climate change probability for a given year while multipliers >1 increase the probability of shifting to a different forest zone. We used a similar approach to develop MC2-derived inter-annual wildfire multipliers where we examined how much area burned in a given year, relative to the average for 2010–2100 (see [29] for method details). Through this approach, a coincidence of both wildfire and a large vegetation change multiplier would be needed to greatly increase the likelihood of vegetation change—a high vegetation trend value without fire prohibited change (i.e., inertia).

Because MC2 projections are deterministic for a given climate scenario, we added a stochastic element to the wildfire and vegetation trend values used in our cSTSMs. Following [46], we randomly selected a wildfire and vegetation trend value within a 20-year moving window (+/- 10 years for any given year) for each simulation year and model run. This approach allowed us to maintain the overall climate, vegetation, and wildfire trends projected by MC2 while recognizing the timing of the shift with climate is less exact than modeled by MC2. While it is possible to run our cSTSMs beyond 2100, we felt it was too speculative to extend our simulation length beyond the 90-year period provided by the three GCMs. Without understanding how climate beyond this century might compare to our simulation period, the rate and direction of vegetation and wildfire trends is too uncertain.

Although we ran MC2, and developed a set of cSTSMs assuming no fire suppression, we also developed another set of cSTSMs where we halved the projected trend in MC2 area burned. Reducing future area burned by 50% does not mean humans will only be able to suppress half of future wildfires; future fire suppression success may continue to approximate current levels, but future area burned may nonetheless increase with warming, lower snowpack, and lower fuel moisture. While reducing future area burned in half is arbitrary, the objective of the scenario was not intended to represent an outcome whose actual future success cannot be known. Rather, running STSMs with a lowered future area burned helps illustrate the potential role of fire suppression as a management tool in a high-inertia landscape under a changing climate.

### Model scenarios and runs

We ran each cSTSM under four climate scenarios (CSIRO, HadGEM, NorESM and no climate change) assuming no vegetation management and either no reduction in area burned or 50% wildfire reduction due to suppression efforts. No other forest management was included in any model. We ran each model for 90 time steps (2010–2100) and 100 simulation iterations. We used an existing ruleset [29] to classify a 2010 map of vegetation (https://lemma.forestry.oregonstate.edu/data) to provide initial forest structure conditions for our model runs.

Here we present MC2 and cSTSM region-wide trends for forest zones and wildfire. We also present cSTSM results for two key structural stages: early- and late-seral conditions, which support exceptional diversity for threatened species of concern [32, 33]. Early-seral states included all grass/forb and post-disturbance conditions. We defined late-seral conditions as areas with average quadratic mean diameters > ~51 cm, multi-storied (>1 canopy layer), and with moderate to high canopy cover (>40%).

## Results

### Wildfire projections

**Fig 3:**
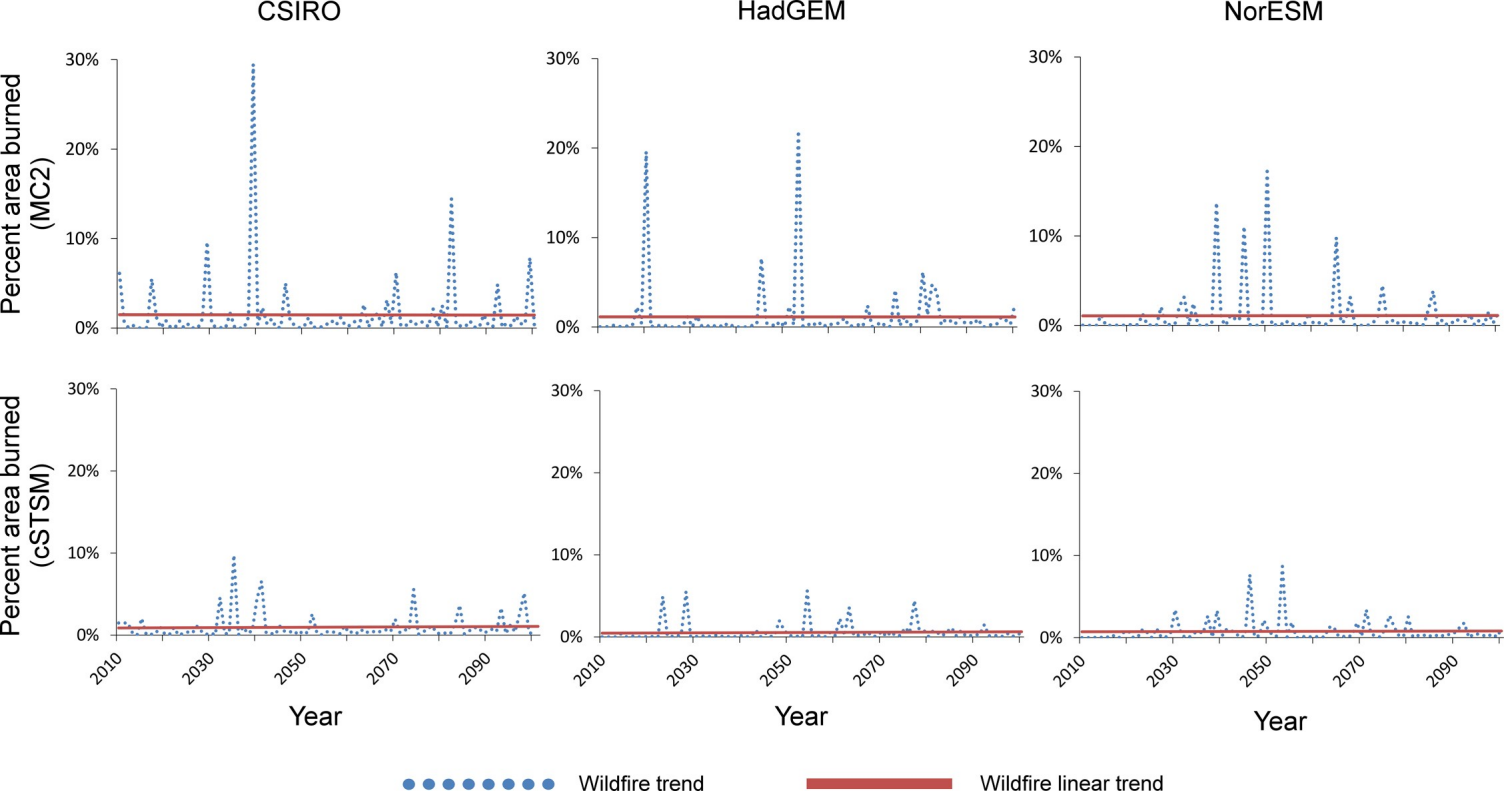
Projected wildfire trends by MC2 and a randomly selected cSTSM simulation iteration. Differences in wildfire projections between MC2 and cSTSMs largely reflect distinct model assumptions about wildfire. Specifically, MC2 is deterministic in setting burn probabilities/events, whereas the stochastic nature of cSTSMs results in fewer wildfire opportunities (even with a slightly increasing trend in area burned with time under changing climate).

**Fig 4:**
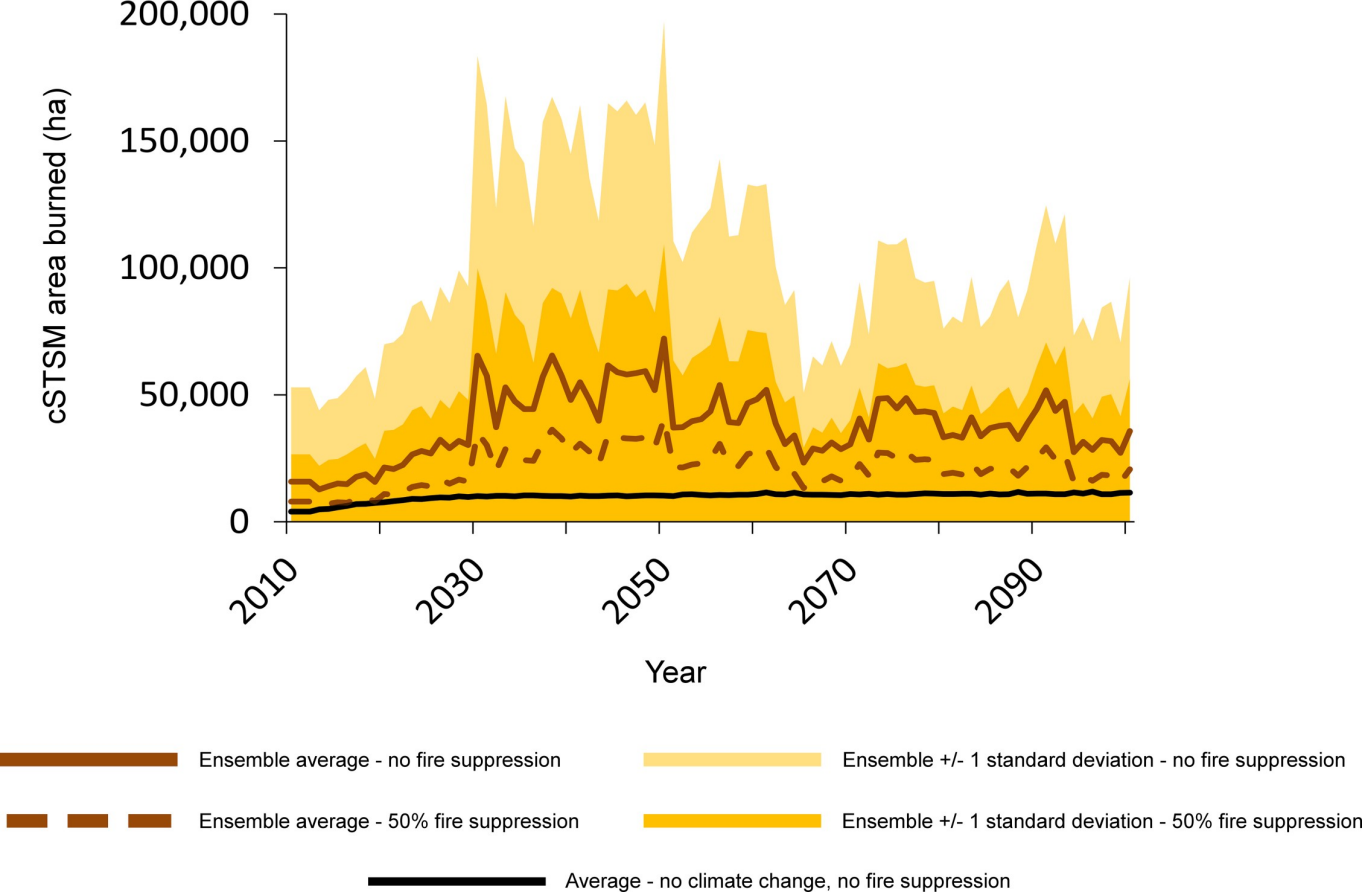
Trends and variation in wildfire, as projected by cSTSMs. Partial fire exclusion assumes a 50% reduction in area burned.

Across climate projections, wildfire increased in western Washington in all MC2 and cSTSM model runs. Historical burn rates were similar between the two modeling approaches (0.24–0.3% of study area annually); however, projected future increases were far smaller under the cSTSMs (mean future burn rate of 0.7%) than in MC2 (mean future rate of 1.2%) (Table 2, Fig 3). Including fire suppression greatly lengthened fire rotations. The year-to-year variation in our cSTSM fire trends was large, varying from zero in some years to tens of thousands of hectares in other years (Fig 4).

**Table 2:** Average annual percent area burned and fire rotation projected by each model under different climate scenarios.

Model	Historical	CSIRO	HadGEM	NorESM	Future average
MC2					
Average percent annually burned	0.30%	1.50%	1.10%	1.10%	1.20%
Fire rotation	333 yrs	67 yrs	91 yrs	91 yrs	83 yrs
cSTSM–no future fire suppression					
Average percent annually burned	0.20%	0.90%	0.50%	0.80%	0.70%
Fire rotation	416 yrs	111 yrs	200 yrs	125 yrs	143 yrs
cSTSM–with future fire suppression					
Average percent annually burned	0.20%	0.50%	0.30%	0.40%	0.40%
Fire rotation	416 yrs	200 yrs	333 yrs	250 yrs	250 yrs

Note our suppression values assume a 50% reduction in area burned from fire suppression efforts.

### Vegetation projections

**Fig 5:**
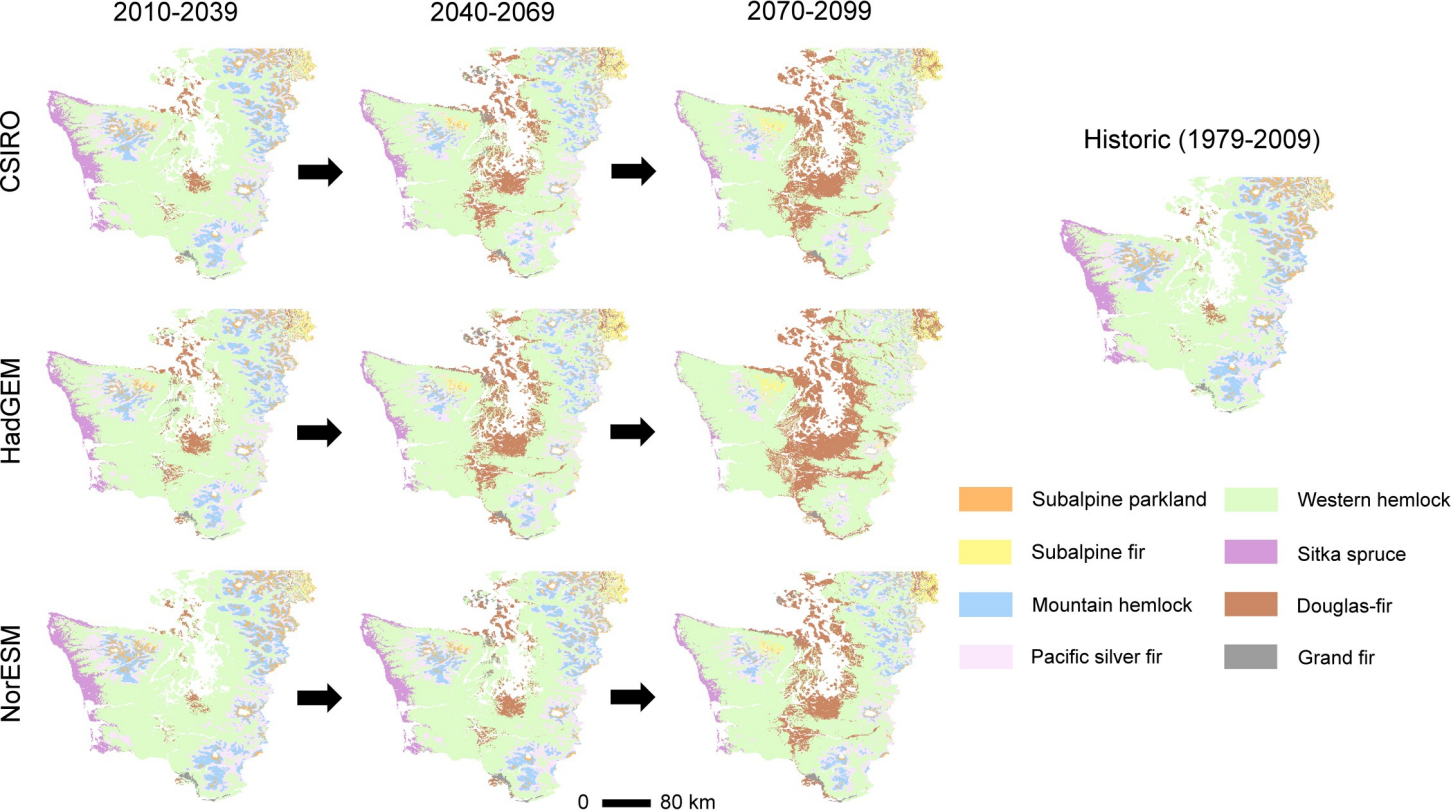
MC2 forest zone projections across three GCMs (30-year modal values). Maps represent vegetation potential based on biophysical properties and climate.

MC2 classified the entire western Washington study area as a forest biome under all climate futures, suggesting forest cover can be maintained through the end of the century. However, potential vegetation conditions steadily departed from historical conditions over the course of the century for all three climate projections in MC2 (Fig 5). The Douglas-fir zone, currently mostly restricted to the rain shadow of the Olympic Peninsula and small portions of the Puget Lowlands, expanded across some of the lowest elevation portions of the study extent, with corresponding losses of other forest zones, mostly the western hemlock zone. The western hemlock zone expanded into the Sitka spruce and Pacific silver fir zones. The remaining zones were generally projected to shift upward in elevation. The climatically-suitable conditions for the subalpine parkland zone largely disappeared. Based on 30-year modal vegetation values, agreement in the spatial distribution of vegetation zones areas across all three MC2 projections and the historical period (1979–2009) declined over time from 73% (2010–2039), to 59% (2040–2069 time period), to 38% (2070–2099), on average (Fig 5). The 30-year modal vegetation projections themselves diverged less from each other; the agreement among the three future MC2 vegetation projections declined from 81%, to 76%, to 70% for the same three time periods.

**Fig 6:**
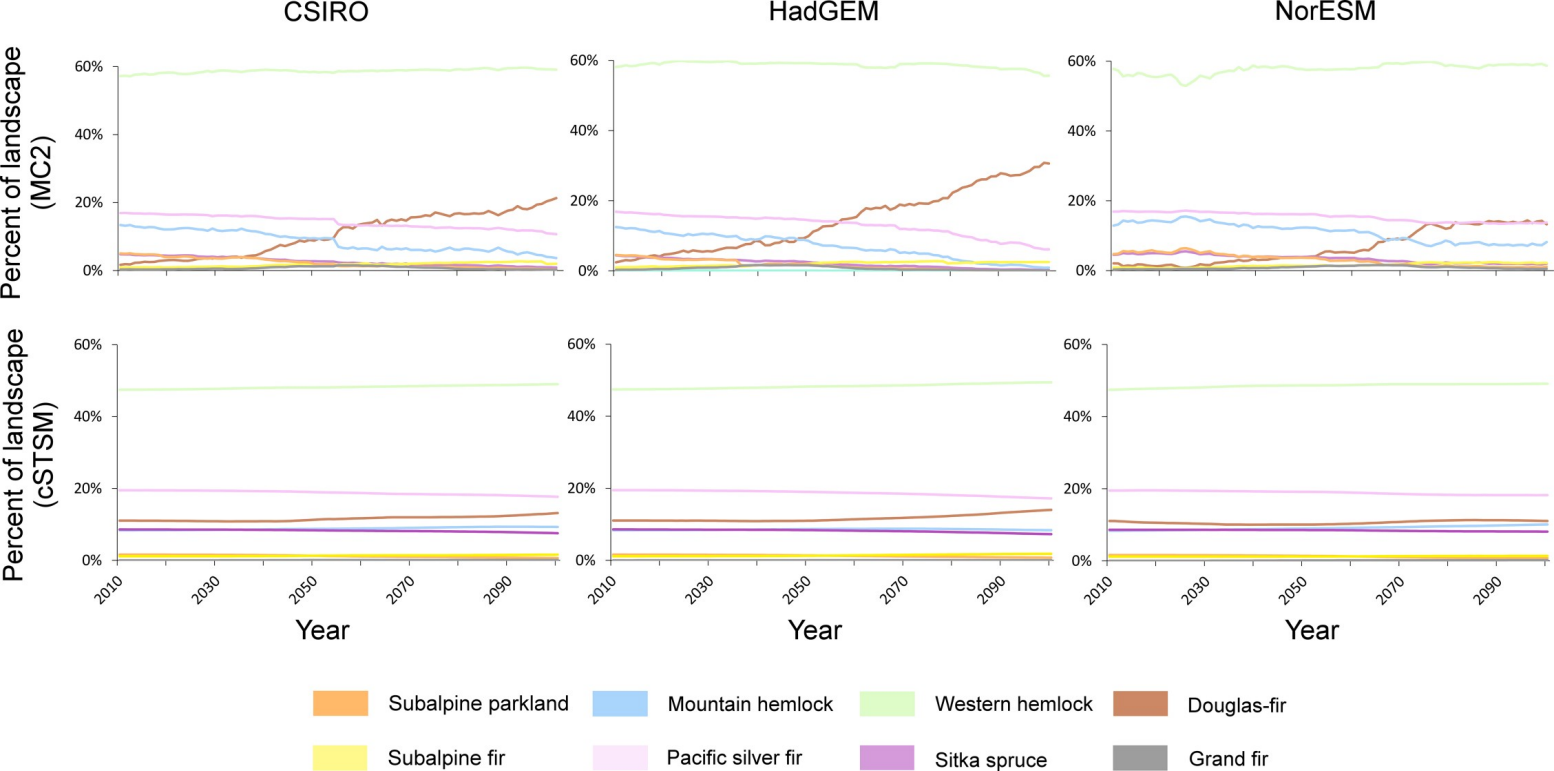
Projected MC2 (top row) and average cSTSM (bottom row) forest zone trends. Differences between MC2 and cSTSM trends reflect differing assumptions of landscape inertia: MC2 shows incremental change in *potential* distribution without accounting for inertia effects, while cSTSM only allows *actual* change to occur after high-severity fires.

**Fig 7:**
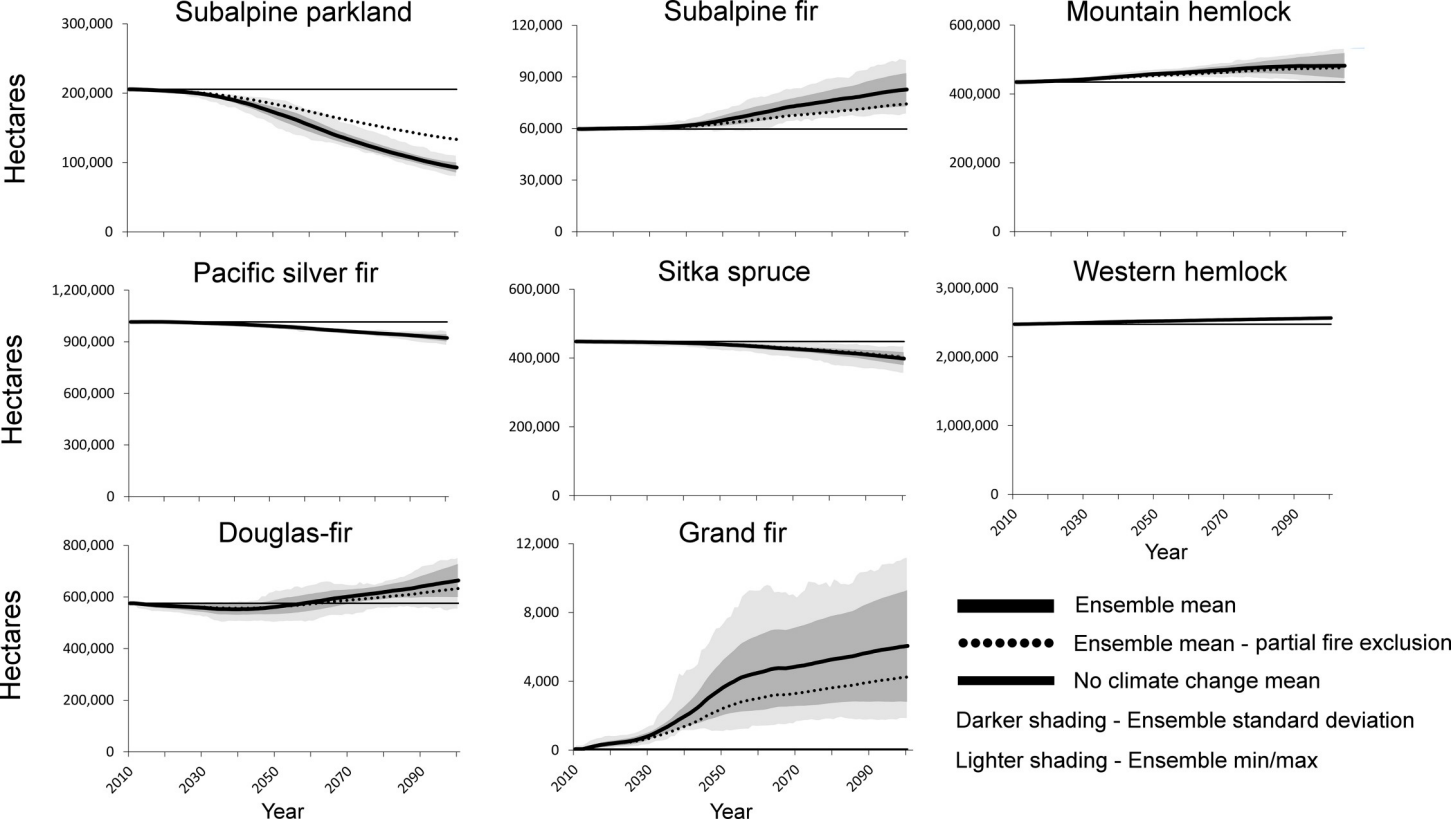
Trends and variation in projected cSTSM forest zones. Partial fire exclusion assumes a 50% reduction in area burned. Note y-axes have different scales and some lines have near-complete overlap.

In contrast to MC2, cSTSMs without fire suppression generally projected forest zone stability across western Washington, in part because of the rarity of fire in the system even under warmer conditions (Figs 6 and 7). The Douglas-fir zone still expanded and the Sitka spruce zone contracted, but changes in area of both zones were minor relative to trends projected by MC2 (Fig 6). Similarly, declines in subalpine parkland were muted compared to those in MC2, and the zone persisted through the century. In absolute numbers, variation across model iterations within a forest zone was relatively small (Fig 7). With fire suppression, vegetation trends were similar, although the amount of decline in a zone tended to be less relative to the no-suppression cSTSM simulations (Fig 7). However, this dampening effect in vegetation shifts was non-linear, with a reduction in wildfire having the greatest effect at the highest (subalpine parkland and subalpine fir) and lowest elevations (Douglas-fir).

**Fig 8:**
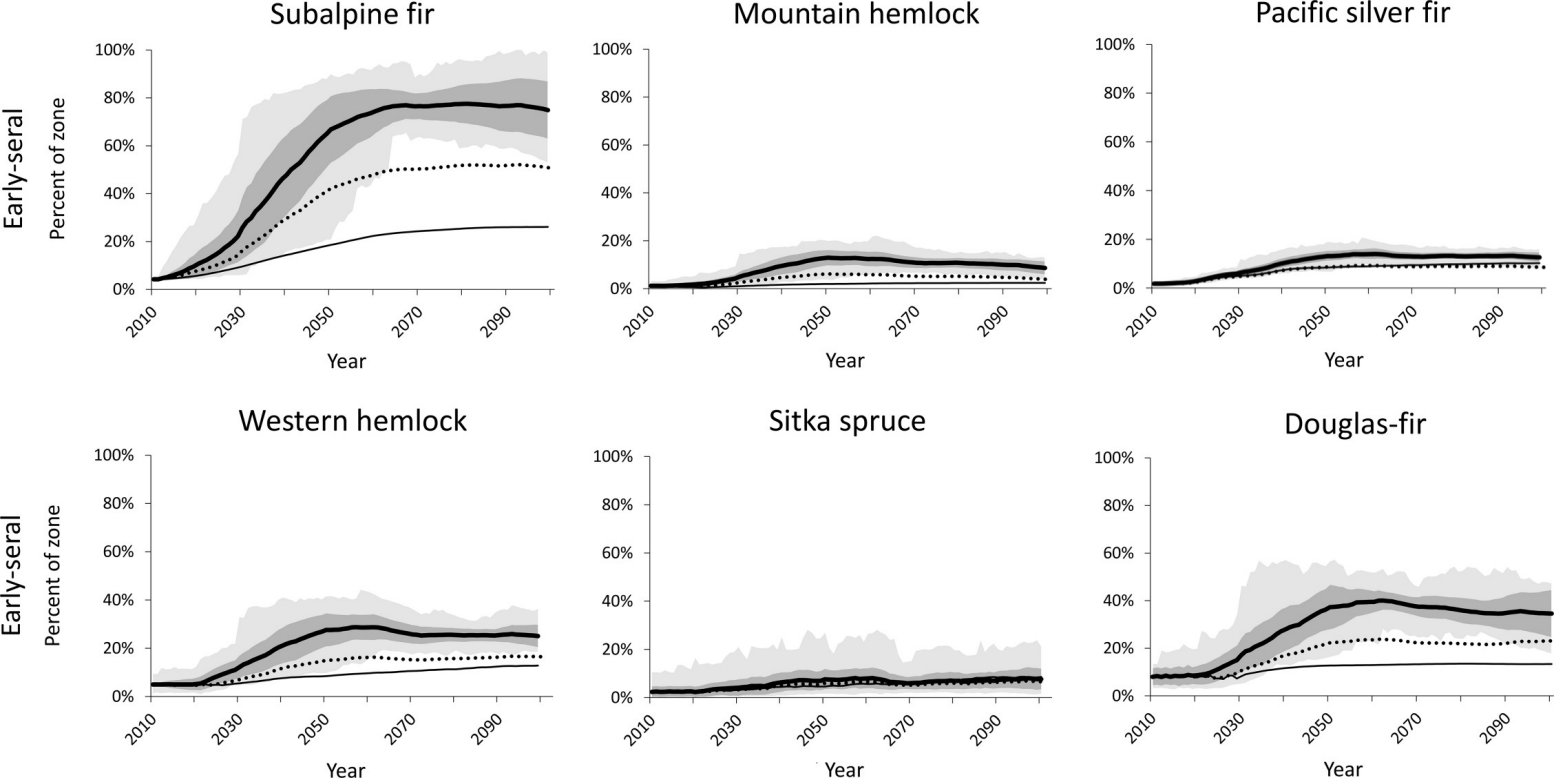
Projected trends and variation in early-seral conditions under difference climate and fire exclusion assumptions. Partial fire exclusion assumes a 50% reduction in area burned. Note y-axes have different scales and some lines have near-complete overlap. See Fig 7 for legend.

**Fig 9:**
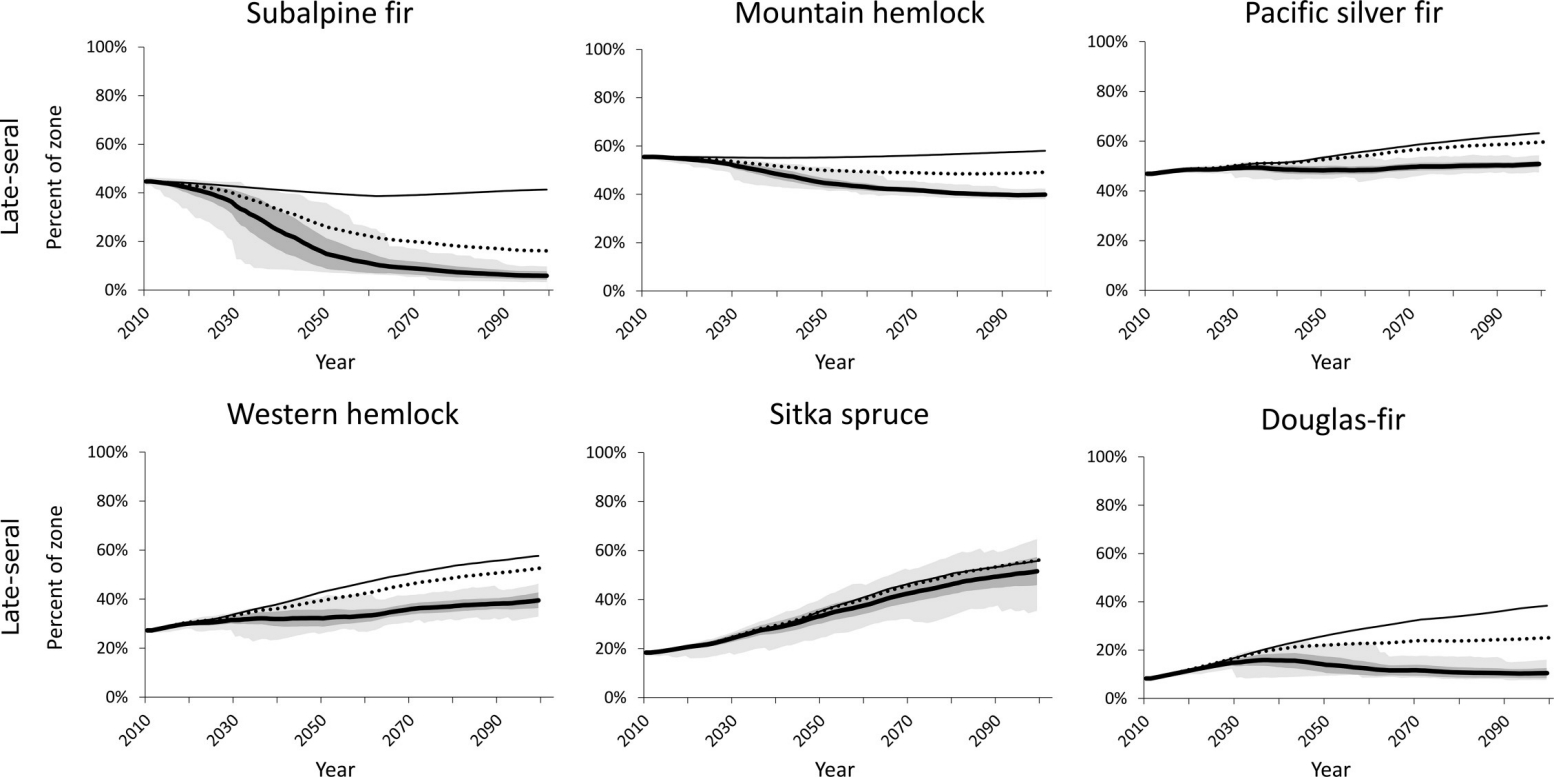
Projected trends and variation in late-seral conditions under difference climate and fire exclusion assumptions. Partial fire exclusion assumes a 50% reduction in area burned. Note y-axes have different scales and some lines have near-complete overlap. See Fig 7 for legend.

Although forest zones as modeled by our cSTSMs remained fairly stable in area with time, greater variation was projected within each forest zone in early-seral and late-seral conditions, depending on climate and wildfire assumptions (Figs 8 and 9). Across western Washington, early-seral area averaged less than 10% of the initial landscape and generally stayed below this level throughout the century under a static climate. With climate change and no fire suppression, early-seral levels increased an additional 2–60%, depending on forest zone (Fig 8). Mitigating the increases in area burned under a changing climate via fire suppression reduced area in early-seral condition by varying amounts, depending on cSTSM-specific fire rotation.

Late-seral area was projected to be greatest assuming no climate change (Fig 9). This increase in late-seral forest can be largely attributed to our initial simulation conditions, which reflect low amounts of late-seral forest on the contemporary landscape as a result of historical and current forest management. With changing climate and no fire suppression, late-seral area declined in the two highest-elevation forest zones, remained relatively stable in the Pacific silver fir zone, and continued to increase at lower rates (compared to no climate change) in the western hemlock and Sitka spruce zones. Reducing area burned by 50% increased late-seral retention in all forest zones, although area in the late-seral condition still declined in the subalpine fir and mountain hemlock zones because of lower growth rates in combination with increasing wildfire. The Subalpine parkland zone was excluded from the late-seral analysis because such structural conditions have less relevance in that zone and are not represented in the cSTSM forest zone model.

## Discussion

### Landscape inertia and vegetation models

By including landscape inertia, our results suggest the maritime forests of the Pacific Northwest may remain relatively stable with climate change over the 21^st^ century in the absence of stand-replacing disturbances such as wildfire. Examining vegetation potential, as in MC2, provides a useful initial perspective, in part because such projections can help land managers evaluate the likelihood of success in attaining current and future social, economic, and ecological goals (Fig 5). Furthermore, recent MC2 work has illustrated the importance of simulating wildfire, climate, and vegetation dynamically (rather than keeping any of those variables static) to better capture how wildfire and vegetation can influence each other through time [51]. However, a consequence of this modeling approach is that MC2 generally projects more rapid changes in vegetation than is likely realistic (Figs 5 and 6). In contrast, including landscape inertia in the cSTSMs prevented widespread vegetation shifts until a major disturbance event occurred. This difference in vegetation shifts by either including or ignoring inertia was also found by [52] using a spatially explicit wildfire model. We believe that including landscape inertia is more consistent with the biophysical processes in the study area, since broad-scale changes in vegetation communities are most likely after the infrequent stand-replacing disturbances that characterize some systems [4, 21, 22].

In addition to differences in the models themselves, MC2 and cSTSM trends often diverged in this landscape because fire rotations are naturally long with inherently limited opportunities for stand-replacing wildfire. For example, an eventual doubling or tripling of wildfire in a western hemlock zone with a 400-year fire rotation is not trivial, but the consequences from such a change in fire frequency are unlikely to be fully expressed in one century. In contrast, similar modeling efforts in a frequent-fire landscape east of the Cascade Mountains [29, 53] illustrated more parallel trends between the MC2 and cSTSM models during this century because opportunities for change were more common (i.e., low inertia) with a more frequent disturbance regime. In high-inertia forests, the time horizon over which disturbances will allow the system to shift are so long (perhaps multiple centuries, even under warmer conditions) that the prevailing climatic conditions under which many of those shifts will occur are beyond what most climate models can predict with any certainty. This will present a fundamental challenge to setting expectations and managing for long-term change in these systems.

### Characterizing wildfire in a high-inertia landscape

Changes in disturbance regimes, particularly fire, will play a key role in forest response to future climate change in both low- and high-inertia landscapes. But our results suggest the frequency, and therefore opportunities for such events, will vary widely. Even within a high-inertia landscape, ranges in projected area burned are broad, in part reflecting differences in climate projections and long fire rotations (Table 2). For example, while HadGEM was the hottest and driest scenario simulated, the more moderate CSIRO scenario projected more area burned (Table 2). This result is counter-intuitive when HadGEM and CSIRO temperature and precipitation projections are viewed on an annual basis, but fire is dependent on spring and summer moisture and temperature. Viewed seasonally, spring and summer projections under each GCM are not very different, with CSIRO actually being even drier than HadGEM during the important late summer period when fires are most likely.

Both our MC2 and cSTSM wildfire projections without fire suppression are within the ~160–1170% range projected in other studies using the MC model across western Washington and Oregon [47, 54]. Incorporating fire suppression, our cSTSM fire projections results tended to be outside, or span, the lower ranges from other studies (an increase of 110–210%). The ecological significance of these projected changes in fire vary with assumptions about future fire suppression. A reduction in the fire rotation to an average of 83 years, as projected by MC2 without fire suppression, would drastically change the sustainability of the myriad values currently associated with western Washington forests. Conversely, shortening rotations from 417 years (across all forest zones) to an average of 250 years in our cSTSMs, assuming fire suppression and landscape inertia, has fewer socio-ecological consequences.

Taken together, the only near-certainty from all wildfire projections, both in this study and other studies, is that future wildfire risk will increase in western Washington. Even without fire projections, this conclusion can be reached based on three conditions often associated with fires during the 20^th^ century in this region: unseasonably dry summer conditions, an ignition source (typically human), and synoptic east wind events bringing already warm and dry continental air toward the ocean [35, 55]. While there does not appear to be published projections about the frequency of future synoptic east wind events under a changing climate, summers will likely become warmer and drier [56]. In combination with future regional population increases (i.e., ignition sources), future wildfires will become more likely.

The degree to which fire suppression, the default policy throughout much of the study region, will be successful in a changing climate further complicates wildfire projections, as severe fire weather and dry fuels will likely become more common [57]. However, fire suppression success will likely have important implications for vegetation shifts. In maritime coniferous forests, with historical fire return intervals of hundreds of years, suppressing fire may stall vegetation shifts that may otherwise occur under a changing climate after severe disturbances [3, 4, 21, 22]. The cSTSM results indicate vegetation shifts will be uneven, suggesting fire suppression effects will vary by sensitivity of forest zone to changes in climate (further discussed below).

### Potential effects of climate change on early- and late-seral structural conditions

With an increase in area burned across climate scenarios and no mitigation of that increase via fire suppression, the amount of area in an early-seral condition increased over time in the cSTSM simulations (Fig 8). In some forest zones, such as Douglas-fir, some of the increase in early-seral area was brought about when a formerly wetter vegetation type burned with stand-replacement severity and converted to a drier early-seral vegetation type under the new climatic conditions. Reducing area burned with fire suppression moderated increases in early-seral area, but there were still increases through the century in most forest zones. Increases in early-seral habitats would benefit wildlife species that utilize them, such as ungulates, Lepidopterans, and some songbirds [33, 58].

In contrast to early-seral habitat, late-seral conditions generally declined in the high elevation types (i.e., subalpine fir and mountain hemlock) with changing climate, but increased slightly (western hemlock, Douglas-fir, and Pacific silver fir) or substantially (Sitka spruce) in the lower elevation forest zones (Fig 9). Loss of late-seral habitat in the high-elevation types can be attributed to a combination of increasing fire frequency and loss of area in these zones. At the lowest elevations, in the Sitka spruce zone, very long fire rotations (1,000 years historically) precluded loss and allowed increase of late-seral habitat. In the other lower-elevation types where forest management occurs, the absence of forest management in the models allowed forest regrowth and development of late-seral structure, despite increased fire frequency with climate change. However, forest management (e.g., rotation harvests) will likely continue in the future, and the combination of increased fire frequency and forest management may result in loss or minimal gain of late-seral structure. Fire suppression may temporarily mitigate these losses, as late-seral habitat area was generally higher in the fire suppression scenario. Thus, fire suppression (until it eventually fails) may be a viable climate adaptation strategy in these forest types to help restore and maintain late-seral structure for species of concern, such as the Northern Spotted Owl (*Strix occidentalis caurina*) [3].

### Potential effects of climate change on vegetation composition

Vegetation zones remained relatively stable in area through the 21^st^ century in cSTSM simulations, and western hemlock remained the dominant forest zone across the landscape. This modeled inertia is a reflection of long fire rotations, running the models only through the end of the century, and the relative persistence of forest zones following disturbance. But high-elevation types such as subalpine parklands could be one of the exceptions to zone stability in western Washington. The projected declines in this zone are consistent with projected contractions of high-elevation forest types in other modeling studies in the region (e.g., [47, 54]). However, the dominant limiting factors to tree establishment at the highest elevations may not change substantially before the end of the century, suggesting that high elevation zones may have more inertia than indicated in most modeling studies.

Though the climatic changes we assessed may not result in dramatic shifts in total area of many vegetation zone in western Washington, shifts in species composition within these zones are possible, particularly following a stand-replacing disturbance (absent subsequent planting). Douglas-fir, with higher drought tolerance and fire resistance than many co-occurring species such as western hemlock and western redcedar [8], will likely become more dominant at lower elevations, as illustrated by the expansion of the Douglas-fir zone in the MC2 and cSTSM simulations, particularly in the Puget Lowlands and northeastern (rain shadow) portion of the Olympic Peninsula. This trend is consistent with other analyses. For example, [59], using LANDIS-II, found increasing temperatures and decreasing summer precipitation may impede the typical seral transition from Douglas-fir to western hemlock in the Puget Lowlands. The expansion of Douglas-fir and lodgepole pine at mid- to low elevations in the dry northeastern portion of the Olympic Peninsula was projected by [60] using a gap model (ZELIG). Paleoecological records also support the potential for future expansion of these species. During the warm and dry Holocene period circa 10,000–6,000 BP, red alder, Douglas-fir, and lodgepole pine were abundant in forests of primarily western hemlock and spruce at lower elevations on the Olympic Peninsula [34, 35, 61, 62]. The abundance of these species in the Pacific Northwest has been associated with higher fire frequency [63, 64]. A study in the North Cascade Range also found increased abundance of lodgepole pine, in association with high fire frequency, circa 10 500–8 000 BP [64]. However, severe moisture limitations may stress or eliminate even relatively drought-tolerant species like Douglas-fir and lodgepole pine from the driest sites [4].

The locations of vegetation zones will also likely shift with changing climate, even if the assemblages that compose the zones persist. MC2 simulates the western hemlock zone moving upward in elevation into portions of what is currently the Pacific silver fir zone, which in turn moves upward into portions of what is currently the mountain hemlock zone in the Olympic and Cascade Mountains. Similarly, the subalpine fir zone moved into what is currently the subalpine parkland zone. These types of shifts have occurred during warming in the past. For example, during a warmer period in the 19^th^ century, western hemlock became dominant in areas where Pacific silver fir and mountain hemlock were dominant on Mount Rainier in the Washington Cascade Mountains [65].

Broadly, while the forests of western Washington have low fire resistance, these forests have been highly resilient to stand-replacing wildfires in the past [3], meaning they could historically experience a severe fire without shifting to an alternative ecosystem state over the long-term (adapted from [66]). With a future increase in area burned, we would continue to expect low resistance (which can be enhanced via fire suppression) but lower resilience than in the past at some point. When that climatic threshold in resilience will be crossed is uncertain, and whether or not we continue to suppress fires, the changes are likely to be abrupt and extensive due to the nature of wildfire in these systems [3].

### Vegetation impact models: Methodological considerations

Several types of dynamic vegetation models, such as LPJ [67] and BIOME [68], utilize the “climate envelope” concept, where vegetation only exists within its climate tolerance [69]. These envelopes are usually determined from current species distributions [69], and are thus limited by available data. Similarly, the envelopes in our models are limited by data. For example, in the case of the largest predicted change in climate of 6°C and plus or minus 10 percent in precipitation, there is no place in North America with a westerly flow of maritime air into a mountain range which could be called a surrogate for validating such predicted future vegetation. [44] also found precipitation and fog effect to have a stronger influence in modeling vegetation distribution and response in the gradient model than temperature and cold air drainage. Ecologically, the relative influence of both variables suggests many trees in the region are relatively insensitive to changes in temperature, but sensitive to changes in precipitation or soil moisture. However, no long-term trend in precipitation is projected for the region (but seasonal precipitation changes are expected) [56]. Although the GCMs were in part selected to encompass a range of temperature and precipitation futures, it is possible a different selection of GCMs would have resulted in different MC2 projections given gradient model and plant species sensitivities to precipitation.

A possibility for future research is the incorporation of the regeneration niche into MC2. The regression equations developed by [44] are based on adult trees. Assuming the regeneration envelope of a species is narrower than that of a mature tree, incorporating information on individual species’ regeneration niche into MC2 would provide a different picture of future potential vegetation. For example, it might be possible to incorporate the rulesets used in the regeneration niche model TACA [70] into MC2, thereby examining the probability of individual species regeneration within a forest zone under different climate projections. This information could also help managers begin to identify if, when, and how current seed stock and seedlings might need to change given different climate futures.

Our cSTSM projections depict gradual changes in forest zones and structural attributes. This gradual change is partly an artifact of running hundreds of model iterations, which (on average) results in some amount of change occurring for any given year. Because these forest zones have largely evolved under a stand-replacing disturbance regime, we are more likely to witness abrupt changes in these systems following stand-replacing events, rather than the gradual changes projected. Furthermore, our models implicitly assume an available seed source following stand-replacing events. Depending on the spatial extent of future fires, it is possible for a delay in succession if seed sources are not present. Nonetheless, the joint MC2-cSTSM approach identifies likely long-term trends.

We also recognize our models ignore other possible drivers of vegetation change, especially biotic drivers such as insects and disease. However, there is still too much scientific uncertainty around the possible changes in biotic disturbances to adequately parameterize the model [71]. Unlike individual species process-based models, our cSTSMs also implicitly assume the plant communities and successional rates currently associated with our modeled forest zones will have relevance under a changing climate. However, both rates of succession and plant communities are likely to change at an unknown point with climate.

Lastly, evidence is lacking in the role of surface and mixed-severity fires in the study region, where fire regimes are nominally ‘stand-replacing’ [72].Consequently, all forest zone STSMs, other than the Douglas-fir forest zone, only include stand-replacing wildfire transitions. We recognize this is an over-simplification of wildfire regimes in western Washington (as most fire events span all severities at a coarse scale). However, evidence suggests non-stand-replacing fire becomes more common south of Washington [72] and both low- and mixed severity fire are not generally considered to reset succession in these systems [73, 74]. Our vegetation zone and late-seral projections would therefore remain unchanged even if non-stand-replacing wildfire transitions were included in all cSTSMs.

## Conclusions

Overall, the inertia of these maritime forests may give land managers a longer window of opportunity to mitigate and prepare for current and future climate change effects on vegetation. Change will occur, but in the absence of a major stand-replacing disturbance event, it will likely occur at a slower pace relative to more frequently-disturbed and less-buffered systems. Ensuring that proactive policies are in place to successfully respond to a stand-replacing disturbance, and developing adaptation strategies and plans, are initial steps to successfully adapt to a changing climate (e.g., [3, 75]). In contrast to frequently-disturbed forests where active management is often promoted prior to a disturbance, it could be mainly after stand-replacing events that managers have an opportunity to adapt large areas to future climate conditions in high-inertia forests.

## Supporting information

S1 Table Similarity in mapped forest zones between the species distribution model (SDM) developed by [1] and the MC2 dynamic global vegetation model for the 1979–2009 period. (DOCX)

S2 Table Ecological description of each MC2 forest zone and state-and-transition simulation model (STSM) used in the analysis. (DOCX)

## References

[1] Barbero R, Abatzoglou JT, Larkin NK, Kolden CA, Stocks B. Climate change presents increased potential for very large fires in the contiguous United States. International Journal of Wildland Fire. 2015; 24(7):892–99.

[2] Westerling AL, Turner MG, Smithwick EA, Romme WH, Ryan MG. Continued warming could transform Greater Yellowstone fire regimes by mid-21st century. Proceedings of the National Academy of Sciences. 2011; 108(32):13165–70. 10.1073/pnas.1110199108 PMC3156206 21788495

[3] Halofsky JS, Donato DC, Franklin JF, Halofsky JE, Peterson DL, Harvey BJ. The nature of the beast: examining climate adaptation options in forests with stand‐replacing fire regimes. Ecosphere. 2018; 9(3).

[4] Littell JS, Oneil EE, McKenzie D, Hicke JA, Lutz JA, Norheim RA, et al Forest ecosystems, disturbance, and climatic change in Washington State, USA. Climatic Change. 2010; 102(1–2):129–58.

[5] Millar CI, Stephenson NL. Temperate forest health in an era of emerging megadisturbance. Science. 2015; 349(6250):823–26. 10.1126/science.aaa9933 26293954

[6] Schoennagel T, Veblen TT, Romme WH. The interaction of fire, fuels, and climate across Rocky Mountain forests. AIBS Bulletin. 2004; 54(7):661–76.

[7] Keane RE, Agee JK, Fulé P, Keeley JE, Key C, Kitchen SG, et al Ecological effects of large fires on US landscapes: benefit or catastrophe? International Journal of Wildland Fire. 2009; 17(6):696–12.

[8] Agee JK. Fire ecology of Pacific Northwest forests. Island press. 1993.

[9] Romme WH. Fire and landscape diversity in subalpine forests of Yellowstone National Park. Ecological Monographs. 1982; 52(2):199–21.

[10] Brubaker LB. Responses of tree populations to climatic change. Vegetatio. 1986; 67(2):119–30.

[11] Abatzoglou JT, Rupp DE, Mote PW. Seasonal climate variability and change in the Pacific Northwest of the United States. Journal of Climate. 2014; 27(5):2125–42.

[12] Mote PW. Climate-driven variability and trends in mountain snowpack in western North America. Journal of Climate. 2006; 19(23):6209–20.

[13] Mantua N, Tohver I, Hamlet A. Climate change impacts on streamflow extremes and summertime stream temperature and their possible consequences for freshwater salmon habitat in Washington State. Climatic Change. 2010; 102(1–2):187–23.

[14] Luce CH, Abatzoglou JT, Holden ZA. The missing mountain water: Slower westerlies decrease orographic enhancement in the Pacific Northwest USA. Science. 2013; 342(6164):1360–4. 10.1126/science.1242335 24292627

[15] Luce CH, Holden ZA. Declining annual streamflow distributions in the Pacific Northwest United States, 1948–2006. Geophysical Research Letters. 2009; 36(16).

[16] Dalton MM, Mote PP, Snover AK. Climate change in the Northwest: implications for our landscapes, waters, and communities Island Press 2013.

[17] Stavros EN, Abatzoglou J, Larkin NK, McKenzie D, Steel EA. Climate and very large wildland fires in the contiguous western USA. International Journal of Wildland Fire. 2014; 23(7):899–14.

[18] Turner MG, Donato DC, Romme WH. Consequences of spatial heterogeneity for ecosystem services in changing forest landscapes: priorities for future research. Landscape ecology. 2013; 28(6):1081–97.

[19] Keppel G, Van Niel KP, Wardell‐Johnson GW, Yates CJ, Byrne M, Mucina L, et al Refugia: identifying and understanding safe havens for biodiversity under climate change. Global Ecology and Biogeography. 2012; 21(4):393–04.

[20] Millar CI, Stephenson NL, Stephens SL. Climate change and forests of the future: managing in the face of uncertainty. Ecological applications. 2007; 17(8):2145–51. 18213958 10.1890/06-1715.1

[21] Crausbay SD, Higuera PE, Sprugel DG, Brubaker LB. Fire catalyzed rapid ecological change in lowland coniferous forests of the Pacific Northwest over the past 14,000 years. Ecology. 2017; 98(9):2356–69. 10.1002/ecy.1897 28500791

[22] Gavin DG, Brubaker LB, Greenwald DN. Postglacial climate and fire‐mediated vegetation change on the western Olympic Peninsula, Washington (USA). Ecological Monographs. 2013; 83(4):471–89.

[23] Stine P, Hessburg P, Spies T, Kramer M, Fettig CJ, Hansen A, et al The ecology and management of moist mixed-conifer forests in eastern Oregon and Washington: a synthesis of the relevant biophysical science and implications for future land management Portland, OR: USDA Forest Service Pacific Northwest Research Station; 2014 p. 254.

[24] Iverson LR, McKenzie D. Tree-species range shifts in a changing climate: detecting, modeling, assisting. Landscape Ecology. 2013; 28(5):879–89.

[25] Guisan A, Thuiller W. Predicting species distribution: offering more than simple habitat models. Ecology letters. 2005; 8(9):993–09. 10.1111/j.1461-0248.2005.00792.x 34517687

[26] Prentice IC, Bondeau A, Cramer W, Harrison SP, Hickler T, Lucht W, et al Dynamic global vegetation modeling: quantifying terrestrial ecosystem responses to large-scale environmental change In: Canagell JG, Pataki DE, Pitelka LF, editors. Terrestrial ecosystems in a changing world. Springer Berlin 2007; 175–92.

[27] Daniel CJ, Frid L, Sleeter BM, Fortin MJ. State‐and‐transition simulation models: a framework for forecasting landscape change. Methods in Ecology and Evolution. 2016; 7(11):1413–23.

[28] Yospin GI, Bridgham SD, Neilson RP, Bolte JP, Bachelet DM, Gould PJ, et al A new model to simulate climate‐change impacts on forest succession for local land management. Ecological applications. 2015; 25(1):226–42. 26255370 10.1890/13-0906.1

[29] Halofsky JE, Hemstrom MA, Conklin DR, Halofsky JS, Kerns BK, Bachelet D. Assessing potential climate change effects on vegetation using a linked model approach. Ecological modelling. 2013; 266:131–43.

[30] Case MJ, Lawler JJ. Integrating mechanistic and empirical model projections to assess climate impacts on tree species distributions in northwestern North America. Global change biology. 2017; 23(5):2005–15. 10.1111/gcb.13570 27859937

[31] Franklin J. Moving beyond static species distribution models in support of conservation biogeography. Diversity and Distributions. 2010; 16(3):321–30.

[32] Franklin JF, Spies TA. Composition, function, and structure of old-growth Douglas-fir forests In: Ruggiero LF, Aubry KB, Carey AB, Huff MH, editors. Wildlife and Vegetation of Unmanaged Douglas-fir Forests. Portland, OR: USDA Forest Service Pacific Northwest Research Station; 1991 p. 533.

[33] Swanson ME, Franklin JF, Beschta RL, Crisafulli CM, DellaSala DA, Hutto RL, et al The forgotten stage of forest succession: early‐successional ecosystems on forest sites. Frontiers in Ecology and the Environment. 2011; 9(2):117–25.

[34] Peterson DL, Schreiner EG, Buckingham NM. Gradients, vegetation and climate: spatial and temporal dynamics in the Olympic Mountains, USA. Global Ecology and Biogeography Letters. 1997; 7–17.

[35] Henderson JA, Shaw DC, Peter DH, Lesher RD. Forested plant associations of the Olympic National Forest Portland, OR: USDA Forest Service Pacific Northwest Region; 1989 p. 502.

[36] PRISM Group. Parameter‐elevation Regressions on Independent Slopes Model Climate Mapping System. 2012. Available at: http://www.prism.oregonstate.edu/.

[37] Daly C, Halbleib M, Smith JI, Gibson WP, Doggett MK, Taylor GH, et al Physiographically sensitive mapping of climatological temperature and precipitation across the conterminous United States. International Journal of Climatology: a Journal of the Royal Meteorological Society. 2008; 28(15):2031–64.

[38] Riahi K, Rao S, Krey V, Cho C, Chirkov V, Fischer G, et al RCP 8.5—A scenario of comparatively high greenhouse gas emissions. Climatic Change. 2011; 109(1–2):33.

[39] Rupp DE, Abatzoglou JT, Hegewisch KC, Mote PW. Evaluation of CMIP5 20th century climate simulations for the Pacific Northwest USA. Journal of Geophysical Research: Atmospheres. 2013; 118(19):10884–96.

[40] Drapek R, Kim JB, Neilson RP. The Dynamic General Vegetation Model MC1 over the United States and Canada at a 5-arcminute resolution: model inputs and outputs Portland, OR: USDA Forest Service Pacific Northwest Research Station; 2015 p. 904.

[41] Bachelet D, Lenihan JM, Daly C, Neilson RP, Ojima DS, Parton WJ. MC1: a dynamic vegetation model for estimating the distribution of vegetation and associated ecosystem fluxes of carbon, nutrients, and water Portland, OR: USDA Forest Service Pacific Northwest Research Station; 2001 p. 95.

[42] Parton WJ, Scurlock JM, Ojima DS, Gilmanov TG, Scholes RJ, Schimel DS, et al Observations and modeling of biomass and soil organic matter dynamics for the grassland biome worldwide. Global Biogeochemical Cycles. 1993; 7(4):785–09.

[43] Conklin DR, Lenihan JM, Bachelet D, Neilson RP, Kim JB. MCFire model technical description Portland, OR: USDA Forest Service Pacific Northwest Research Station; 2016 p. 75.

[44] Henderson JA, Lesher RD, Peter DH, Ringo CD. A landscape model for predicting potential natural vegetation of the Olympic Peninsula USA using boundary equations and newly developed environmental variables Portland, OR: USDA Forest Service Pacific Northwest Research Station; 2011 p. 35.

[45] Omernik JM. Ecoregions of the conterminous United States. Annals of the Association of American geographers. 1987; 77(1):118–25.

[46] Creutzburg MK, Henderson EB, Conklin DR. Climate change and land management impact rangeland condition and sage-grouse habitat in southeastern Oregon. AIMS Environmental Science. 2015; 2(2):203–36.

[47] Sheehan T, Bachelet D, Ferschweiler K. Projected major fire and vegetation changes in the Pacific Northwest of the conterminous United States under selected CMIP5 climate futures. Ecological Modelling. 2015; 317:16–29.

[48] Hudiburg T, Law B, Turner DP, Campbell J, Donato D. Duane M. Carbon dynamics of Oregon and Northern California forests and potential land‐based carbon storage. Ecological Applications. 2009; 19(1):163–80. 19323181 10.1890/07-2006.1

[49] Norby RJ, DeLucia EH, Gielen B, Calfapietra C, Giardina CP, King JS, et al Forest response to elevated CO2 is conserved across a broad range of productivity. Proceedings of the National Academy of Sciences. 2005; 102(50):18052–56. 10.1073/pnas.0509478102 PMC1312431 16330779

[50] Halofsky JE, Creutzburg MK, Hemstrom MA. Integrating social, economic, and ecological values across large landscapes Portland, OR: USDA Forest Service Pacific Northwest Research Station; 2014 p. 206.

[51] Syphard AD, Sheehan T, Rustigian-Romsos H, Ferschweiler K. Mapping future fire probability under climate change: Does vegetation matter? PLoS ONE. 2018; 13(8):1–23. 10.1371/journal.pone.0201680 PMC6078303 30080880

[52] Stralberg D, Wang X, Parisien MA, Robinne FN, Sólymos P, Mahon CL, et al Wildfire‐mediated vegetation change in boreal forests of Alberta, Canada. Ecosphere. 2018; 9(3):p.e02156.

[53] Halofsky JS, Halofsky JE, Burcsu T, Hemstrom MA. Dry forest resilience varies under simulated climate‐management scenarios in a central Oregon, USA landscape. Ecological Applications. 2014; 24(8):1908–25. 29185662 10.1890/13-1653.1

[54] Rogers BM, Neilson RP, Drapek R, Lenihan JM, Wells JR, Bachelet D, et al Impacts of climate change on fire regimes and carbon stocks of the US Pacific Northwest. Journal of Geophysical Research: Biogeosciences. 2011;116(G3). 10.1029/2011jg001641 24349901 PMC3859319

[55] Holbrook SH. Burning an empire: the story of American forest fires The Macmillan company 1960.

[56] Mauger G, Casola J, Morgan H, Strauch R, Jones B, Curry B, et al State of knowledge: Climate change in Puget Sound Report prepared for the Puget Sound Partnership and the National Oceanic and Atmospheric Administration. Climate Impacts Group, University of Washington, Seattle 2015; p. 288.

[57] Flannigan MD, Wotton BM, Marshall GA, De Groot WJ, Johnston J, Jurko N, et al Fuel moisture sensitivity to temperature and precipitation: climate change implications. Climatic Change. 2016; 134(1–2):59–71.

[58] Swanson ME, Studevant NM, Campbell JL, Donato DC. Biological associates of early-seral pre-forest in the Pacific Northwest. Forest Ecology and Management. 2014; 324:160–71.

[59] Laflower DM, Hurteau MD, Koch GW, North MP, Hungate BA. Climate-driven changes in forest succession and the influence of management on forest carbon dynamics in the Puget Lowlands of Washington State, USA. Forest Ecology and Management. 2016; 362:194–04.

[60] Zolbrod AN, Peterson DL. Response of high-elevation forests in the Olympic Mountains to climatic change. Canadian Journal of Forest Research. 1999; 29(12):1966–78.

[61] Heusser CJ. Quaternary palynology of the Pacific slope of Washington. Quaternary Research. 1977; 8(3):282–06.

[62] Vegetational Whitlock C. and climatic history of the Pacific Northwest during the last 20,000 years: implications for understanding present-day biodiversity. Northwest Environmental Journal. 1992; 8:5–28.

[63] Cwynar LC. Fire and the forest history of the North Cascade Range. Ecology. 1987; 68(4):791–02.

[64] Prichard SJ, Gedalof ZE, Oswald WW, Peterson DL. Holocene fire and vegetation dynamics in a montane forest, North Cascade Range, Washington, USA. Quaternary Research. 2009; 72(1):57–67.

[65] Dunwiddie PW. A 6000‐year record of forest history on Mount Rainier, Washington. Ecology. 1986; 67(1):58–68.

[66] Walker B, Holling CS, Carpenter SR, Kinzig A. Resilience, adaptability and transformability in social–ecological systems. Ecology and Society. 2004; 9(2):5.

[67] Sitch S, Smith B, Prentice IC, Arneth A, Bondeau A, Cramer W, et al Evaluation of ecosystem dynamics, plant geography and terrestrial carbon cycling in the LPJ dynamic global vegetation model. Global Change Biology. 2003; 9(2):161–85.

[68] Running SW, Hunt ER Jr. Generalization of a forest ecosystem process model for other biomes, BIOME-BCG, and an application for global-scale models In: Ehleringer JR, Field CB, editors. Scaling physiological processes: leaf to globe. Elsevier 1993; 414–58.

[69] Fisher RA, Muszala S, Verteinstein M, Lawrence P, Xu C, McDowell NG, et al Taking off the training wheels: the properties of a dynamic vegetation model without climate envelopes, CLM4. 5 (ED). Geoscientific Model Development. 2015; 8(11):3593–19.

[70] Nitschke CR, Innes JL. A tree and climate assessment tool for modelling ecosystem response to climate change. Ecological Modelling. 2008; 210(3):263–77.

[71] Agne MC, Beedlow PA, Shaw DC, Woodruff DR, Lee EH, Cline SP, et al Interactions of predominant insects and diseases with climate change in Douglas-fir forests of western Oregon and Washington, USA. Forest Ecology and Management. 2018; 409:317–32. 10.1016/j.foreco.2017.11.004 29290644 PMC5746199

[72] Spies TA, Stine PA, Gravenmier RA, Long JW, Reilly MJ. Synthesis of science to inform land management within the Northwest forest plan area Portland, OR: USDA Forest Service Pacific Northwest Research Station; 2018 p. 1020.

[73] Tepley AJ, Swanson FJ, Spies TA. Fire‐mediated pathways of stand development in Douglas‐fir/western hemlock forests of the Pacific Northwest, USA. Ecology. 2013; 94(8):1729–43. 24015517 10.1890/12-1506.1

[74] Nonaka E, Spies TA. Historical range of variability in landscape structure: a simulation study in Oregon, USA. Ecological Applications. 2005; 15(5):1727–46.

[75] Halofsky JE, Peterson DL, O’Halloran KA, Hoffman CH. Adapting to climate change at Olympic National Forest and Olympic National Park Portland, OR: USDA Forest Service Pacific Northwest Research Station; 2011 p. 130.

