# Supplementary material for: Climate change, wildfire, and vegetation shifts in a high-inertia forest landscape: Western Washington, U.S.A
Source: PLoS One. 2018 Dec 20;13(12):e0209490. doi: 10.1371/journal.pone.0209490 (PMC6301671; doi:10.1371/journal.pone.0209490)
Supplement: S1 Table — (DOCX) [file pone.0209490.s001.docx]

**S1 Table. Similarity in mapped forest zones between the species distribution model (SDM) developed by [1] and the MC2 dynamic global vegetation model for the 1979-2009 period**. Each of the three ecoregions noted below were run separately by the MC2 model.

| *MC2 forest zone* | Washington Coast Range Ecoregion | | Washington North Cascades Ecoregion | | Washington West Cascades Ecoregion | |
| --- | --- | --- | --- | --- | --- | --- |
|  | SDM current extent (%) | MC2 current extent (%) | SDM current extent (%) | MC2 current extent (%) | SDM current extent (%) | MC2 current extent (%) |
| **Subalpine parkland** | 3 | 3 | 13 | 12 | 2 | 2 |
| **Subalpine fir** | 1 | <1 | 1 | 1 | <1 | 0 |
| **Mountain hemlock** | 6 | 7 | 17 | 20 | 7 | 13 |
| **Pacific silver fir** | 12 | 13 | 18 | 17 | 19 | 20 |
| **Western hemlock** | 58 | 62 | 48 | 46 | 68 | 61 |
| **Sitka spruce** | 20 | 15 | <1 | 0 | 0 | 0 |
| **Douglas-fir** | <1 | <1 | 1 | 2 | 3 | 2 |
| **Grand fir** | 0 | 0 | 0 | 0 | <1 | 1 |

**References**

1. Henderson JA, Lesher RD, Peter DH, Ringo CD. A landscape model for predicting potential natural vegetation of the Olympic Peninsula USA using boundary equations and newly developed environmental variables. Portland, OR: USDA Forest Service Pacific Northwest Research Station; 2011. p. 35.
