# Supplementary material for: Climate change, wildfire, and vegetation shifts in a high-inertia forest landscape: Western Washington, U.S.A
Source: PLoS One. 2018 Dec 20;13(12):e0209490. doi: 10.1371/journal.pone.0209490 (PMC6301671; doi:10.1371/journal.pone.0209490)
Supplement: S2 Table — (DOCX) [file pone.0209490.s002.docx]

**S2 Table. Ecological description of each MC2 forest zone and state-and-transition simulation model (STSM) used in the analysis.**

| *STSM* | *Model description* |
| --- | --- |
| **Subalpine parkland** | This model applies to the highest elevation areas and represents subalpine parklands, where trees are patchy or dispersed rather than contiguous, often occurring as islands in grassland, shrubland, and alpine tundra. Subalpine fir (*Abies lasiocarpa* (Hook.) Nutt.) is the dominant late-successional tree species in most areas, though other species may be more abundant locally. Due to short growing seasons, regeneration from a grass/forb to a conifer stand is slow and may exceed 50 years. The average fire rotation is 385 years. |
| **Subalpine fir** | This model represents stands of subalpine fir occurring at high elevation with relatively continental climates. It is similar to many subalpine fir, and associated lodgepole pine (*Pinus contorta*), forests found north into British Columbia, Alberta, and east into Idaho and Montana. Smaller areas are located in rain-shadows. Subalpine fir dominates late-successional stands, but Douglas-fir (*Pseudotsuga menziesii*), Engelmann spruce (*Picea engelmannii* Parry ex Engelm.), and other species may be present. Lodgepole pine and western larch (*Larix occidentalis* Nutt.) are often present or abundant in early-successional stands. With an average fire rotation of 208 years, this model has a greater probability of wildfire than the other high elevation models in the region. Insect disturbance by mountain pine beetle and balsam wooly adelgid may occur. |
| **Mountain hemlock** | This model represents stands of mountain hemlock (*Tsuga merensiana*) and lodgepole pine occurring at high elevations. Lodgepole pine may be present in early-successional stands, especially in somewhat more inland or continental areas. Mountain hemlock, sometimes with Alaska cedar (Cupressus nootkatensis D. Don) or Pacific silver fir (*Abies amabilis*), dominates later-successional conditions in which a full range of tree sizes is often represented. Fire rotations vary between 350 and 833 years depending on the model variant used in a region. Mountain pine beetle outbreaks may occur. |
| **Pacific silver fir** | This model represents mix stands of silver fir, Douglas-fir, western hemlock (*Tsuga heterophylla*), and mountain hemlock at middle elevations. This model applies primarily to silver fir plant association groups that are found in intermediate temperature and moisture locations. Growing seasons are short, dry, and cooler than in the western hemlock zone, and summer frosts more common. Winter snow is common, and snow packs can be persistent, especially in openings such as meadows or clearcuts. Fire rotations average 625 years. Wind disturbances occur, but only at low severity. Insect disturbance, including hemlock looper and balsam wooly adelgid, may occur. |
| **Western hemlock** | This model represents stands composed of a mix of western hemlock and Douglas-fir. This model applies primarily to western hemlock and Douglas-fir plant association groups that are found in locations of intermediate temperature and moisture. Wind disturbances occur, but only at low severity. Hemlock looper outbreaks may occur. Average fire rotations used in this analysis varied from 250 – 400 years depending on model variant used within each region. |
| **Sitka spruce** | This model applies to coastal forests of Washington. Outside of sites right on the coast, Sitka spruce (*Picea*  *sitchensis*) rarely dominates any given stand, but may be a scattered component. Western hemlock generally  dominates, with Douglas-fir, western red cedar (*Thuja plicata*), and Sitka spruce as sub- or co-dominants. Dense alder stands may prevail in some locations after severe disturbance. With a 1,000 year fire rotation, fire is extremely rare but high and low severity wind disturbances frequently occur. Hemlock looper outbreaks may also occur. |
| **Douglas-fir** | This model applies primarily to dry temperate to almost continental climates in western Washington. Dominant tree species are Douglas-fir or Lodgpole pine. Pacific madrone, western hemlock and western white pine may also be present. Balsam wooly adelgid outbreaks may occur. Average fire rotations are 200 years and tend to be of mixed-severity. |
| **Grand fir** | This model applies to forested areas drier than the Douglas-fir model. Grand fir is generally the late-successional species on these sites, but Douglas-fir is abundant in most places. Bigleaf maple often occurs, especially following wildfire or timber harvest. Oregon white oak and Pacific madrone are common on some sites. Average fire rotations are 200 years. |
